# Knowledge, Attitude and Practice of Malawian Farmers on Pre- and Post-Harvest Crop Management to Mitigate Aflatoxin Contamination in Groundnut, Maize and Sorghum—Implication for Behavioral Change

**DOI:** 10.3390/toxins11120716

**Published:** 2019-12-09

**Authors:** Seetha Anitha, Takuji W. Tsusaka, Samuel M.C. Njoroge, Nelson Kumwenda, Lizzie Kachulu, Joseph Maruwo, Norah Machinjiri, Rosemary Botha, Harry W. Msere, Juma Masumba, Angela Tavares, Geoffrey M Heinrich, Moses Siambi, Patrick Okori

**Affiliations:** 1 Smart Food Initiative, International Crops Research Institute for the Semi-Arid Tropics (ICRISAT), Patancheru, Hyderabad 502324, India; 2 Eastern and Southern Africa Research Program, International Crops Research Institute for the Semi-Arid Tropics (ICRISAT), Lilongwe P.O. Box 1096, Malawi; takuji.tsusaka@gmail.com (T.W.T.); n.kumwenda@cgiar.org (N.K.); L.Kachulu@cgiar.org (L.K.); J.Maruwo@cgiar.org (J.M.); nmachinjiri@gmail.com (N.M.); h.msere@cgiar.org (H.W.M.); p.okori@cgiar.org (P.O.); 3 Development Strategy and Governance Division, International Food Policy Research Institute, Lilongwe P.O. Box 31666, Malawi; rozbotha@gmail.com; 4 United in Building and Advancing Life Expectations Project, Catholic Relief Services, Private Bag 319, Lilongwe, Malawi; Juma.Masumba@crs.org (J.M.); angela.tavares@crs.org (A.T.); 5 Agriculture Livelihood and Environment, Catholic Relief Services, Lusaka P.O. Box 38080, Zambia; geoffrey.heinrich@crs.org; 6 Eastern and Southern Africa Research Program, International Crops Research Institute for the Semi-Arid Tropics (ICRISAT), Nairobi P.O. Box 39063, Kenya; m.siambi@cgiar.org

**Keywords:** groundnut, maize, sorghum, aflatoxin control, pre- and post-harvest practices, KAP

## Abstract

A knowledge, attitude and practice (KAP) study was conducted in three districts of Malawi to test whether the training had resulted in increased knowledge and adoption of recommended pre- and post-harvest crop management practices, and their contribution to reducing aflatoxin contamination in groundnut, maize and sorghum. The study was conducted with 900 farmers at the baseline and 624 farmers at the end-line, while 726 and 696 harvested crop samples were collected for aflatoxin testing at the baseline and end-line, respectively. Results show that the knowledge and practice of pre- and post-harvest crop management for mitigating aflatoxin were inadequate among the farmers at the baseline but somewhat improved after the training as shown at the end-line. As a result, despite unfavorable weather, the mean aflatoxin contamination level in their grain samples decreased from 83.6 to 55.8 ppb (*p* < 0.001). However, it was also noted that increased knowledge did not significantly change farmers’ attitude toward not consuming grade-outs because of economic incentive incompatibility, leaving potential for improving the practices further. This existing gap in the adoption of aflatoxin mitigation practices calls for approaches that take into account farmers’ needs and incentives to attain sustainable behavioral change.

## 1. Introduction

To address undernutrition, the government of Malawi has been implementing initiatives to increase the production of and improve access to nutritious foods especially legumes such as groundnut (*Arachis hypogaea* L), common bean (*Phaseolus vulgaris* L) and pigeonpea (*Cajanus cajan* L) among others [1]. In Malawi, groundnut is a major crop grown on 390,000 ha [2], mostly by smallholder farmers. Groundnut production offers a lot of benefits to the farmers in terms of improving soil fertility by fixing atmospheric nitrogen and providing an important source of income and food. Groundnut is consumed locally as roasted or boiled kernels, processed into peanut butter, pressed for oil, or ground into powder that is added to dishes or porridge. Groundnut is also a major ingredient in Ready-to-Use Therapeutic Food (RUTF) that is fed to malnourished children [3]. 

The consumption of groundnut, especially when it is not sorted to remove moldy, shriveled, insect-damaged and broken kernels, increases the risk of aflatoxin exposure for consumers [4]. Maize (*Zea mays* L) is the main staple food in Malawi, as well as in many other sub-Saharan African countries, and is also as susceptible to mold infection and aflatoxin contamination as groundnut. Both maize and groundnut are regularly consumed by households, posing the risk of exposure to aflatoxins. Sorghum (*Sorghum vulgare* (L) Moench) is another major staple food crop grown in the Lower Shire valley of Malawi, an area prone to drought and high temperature, which predisposes grain infection by aflatoxin-producing fungi especially *Aspergillus* species.

Aflatoxin contamination can occur both in the field (pre-harvest and initial post-harvest) and under storage facilities (post-harvest). Pre-harvest contamination, however, is more important when crops experience end-of-season drought [5,6]. Moreover, oil and starchy crops, the harvested parts of which develop underground, such as groundnut and Bambara nut (*Vigna subterranea* (L.) Verdc), tend to be at higher aflatoxin contamination risk compared to crops with harvestable parts above ground [7]. Pre-harvest contamination in maize or sorghum occurs when the fungus infects the kernels via airborne conidia that colonize the silk during flowering or when the kernels are damaged from insect feeding. All starchy crops are susceptible to contamination after harvest, especially if they are dried directly on bare soil [7]. 

A study investigating the prevalence of aflatoxin contamination in both groundnut and maize in Malawi reported an incidence of 8% to 21% [8], above the recommended the Food and Drug Administration (FDA) levels of 20 parts per billion (ppb) of aflatoxin [9]. Subsequent studies have reported increased aflatoxin contamination in grain and food products that are available in markets and households [4,8,10,11]. When consumed, aflatoxin-contaminated food results in adverse nutrition and health consequences [12]. Long-term exposure to sub-clinical aflatoxin levels leads to chronic health outcomes such as cancer and has been linked to childhood stunting, whereas acute exposure leads to aflatoxicosis or death, a rarer outcome [12]. Contaminated grain can also adversely impact trade and the broader economy. Malawi for example has lost a significant export market share, especially to lucrative markets in Europe, since 1990, primarily due to aflatoxin contamination of its groundnut grain [8,11]. 

Farmers can mitigate aflatoxin contamination in crops before harvest and at harvest by adopting appropriate agronomic practices such as timely planting, providing supplemental irrigation, water harvesting, applying manure and also through the application of atoxigenic strains of *Aspergillus flavus* [11,13]. Post-harvest mitigation of contamination is achieved through proper drying of produce after harvest, sorting to remove damaged and shriveled kernels, and storage in well aerated facilities, or in hermetic storage bags [14].

Farmers’ knowledge, attitude and practice (KAP) of mitigating aflatoxin contamination may contribute to lowering aflatoxin contamination and improving nutritional, health, and economic impacts. Due to the severity of the aflatoxin contamination challenge in Malawi, several training programs have been undertaken by diverse organizations, albeit with limited success. There are few studies conducted so far in Malawi to understand farmers’ KAP on mold or aflatoxin contamination, [15] especially their attitude toward practicing taught mitigation approaches and its gaps in implementation. Considering the need for designing effective behavioral change tools to enhance the implementation of mitigation efforts, this study aims to: (a) determine the level of KAP on pre- and post-harvest crop management practices on aflatoxin mitigation, (b) determine the impact of training on pre- and post-harvest crop management on aflatoxin levels in crop samples, and (c) identify gaps in farmers’ attitude toward aflatoxin mitigation practices and their impact on aflatoxin levels in crop samples. To our knowledge, this is the first study conducted to understand the impact of training on KAP and aflatoxin contamination levels with a focus on changes in farmers’ knowledge levels. 

## 2. Results

### 2.1. Demographics and Landholding

Thirty percent of the surveyed households were headed by women. The average number of household members was 5.4—among which, nearly 50% were women. The major economic activity was crop production practiced by 81.9%, 96.5% and 97.5% of the households in Blantyre, Chikwawa and Nsanje, respectively. Almost all (98.4%) of the farmers owned land, and 83.8% of the farmers owned livestock. Approximately 50% of the agricultural labor activities were managed by women, which included sowing, weeding, harvesting and drying. However, stripping and shelling of groundnut were dominantly handled by women, which was consistent with the result previously reported by Orr et al. [16]. The farmers’ average landholding was 1.0 hectare (2.4 acres). The farmers allocated more land for maize compared to the other two crops as maize is the main staple food crop. The average land allocation for maize, groundnut and sorghum was 1.74 acre ± 0.12, 0.84 acre ± 0.12 and 1.04 acre ± 0.18, respectively. When it comes to the yield of the crop produce during the baseline crop season 2016/2017, maize yield was relatively high in Blantyre (634.5 kg/acre ± 2907.2), compared to Nsanje (275.6 kg/acre ± 313.1) and Chikwawa (307.2 kg/acre ± 325.6). Groundnut yield was higher in Nsanje (306.8 kg/acre ± 208.3) than in Blantyre (149.3 kg/acre ± 171.7) and Chikwawa (133.8 kg/acre ± 172.7). On the other hand, sorghum yield was relatively high in Chikwawa (211.4 kg/acre ± 174.0) compared to Nsanje (174.3 kg/acre ± 242.0) and Blantyre (87.0 kg/acre ± 93.5).

### 2.2. Knowledge, Attitude and Practice 

Farmers’ knowledge on aflatoxin contamination and pre- and post-harvest crop management for mitigation was limited at the baseline (Table 1), predisposing their produce to aflatoxin contamination. In particular, the proportion of farmers with knowledge of negative health effects of aflatoxin on children and livestock was remarkably low. Although not presented, 16% of the farmers in Blantyre, 9% in Chikwawa and 7% in Nsanje mentioned that they had knowledge about aflatoxin. Farmers’ knowledge on some particular topics in aflatoxin contamination and crop management practices increased from the baseline to end-line. The knowledge increased significantly with respect to three critical knowledge items: (i) aflatoxin contamination causes income loss (*p* = 0.092), (ii) aflatoxin-contaminated grains should not be fed to livestock (*p* = 0.075) and (iii) aflatoxin-contaminated grains reduce livestock productivity (*p* = 0.033). Although the chi-square test was not supportive, a large percentage point increase was observed in knowledge that contamination spreads if the crop produce is not graded. The improvement in knowledge must be due to the training provided by the team during the 2017–2018 cropping season, provided that the assumption of no external influence holds (see Section 5.1).

The focus group discussions (FGDs) revealed that the farmers did not want to grade adequately and did not want to throw away grade out grain, which was due to a concern over losses in income (Table 2). Farmers stated that they consumed, fed to animals and/or sold grade out grains to markets. Similarly, farmers’ attitude toward drying the produce did not change largely. According to the discussions, they feared thefts of produce that was left in the field and also had insufficient space for drying at homestead. However, farmers were positive about adopting mulching and were willing to continue or adopt the practice as it was an affordable practice. 

Table 3 shows the proportion of farmers practicing the grading process for each of the three crops in the three districts, and its change from the baseline to end-line. In Chikwawa, there was a significant increase in grading sorghum (*p* = 0.097). Similarly, in Nsanje there was a significant increase in grading groundnut (*p* = 0.012). On the whole, changes in farmers’ grading practices were present but limited. 

At the baseline, 50.7% of the farmers dried their crop produce in their fields on the soil or on the rooftop of their houses, while 70.9% dried it on a mat or tarpaulin (Table 4). At the end-line, 95.8% of them dried their produce on tarpaulin sheets rather than on bare ground/floors, though the *p*-value for this increase was larger than 0.10. A majority of farmers manually shelled their groundnut, and the proportion further increased toward the end-line. On the other hand, in spite of the training, some of the unfavorable practices did not decrease significantly, including the practice of sprinkling water to ease hand shelling, which is considered to aggravate contamination. The data show a significant reduction in the proportion of farmers consuming grade outs between the baseline and the end-line (*p* = 0.009). There was also a significant increase in the proportion of farmers who threw away grade outs (*p* = 0.090). A small proportion of farmers started storing their grains hermetically toward the end-line. There was a significant increase in the proportion of farmers who stored their produce in a gunny bag/polythene bag between the baseline and end-line (*p* = 0.086). Likewise, the proportion of farmers who stored their grain bags on palates increased between the baseline and end-line (*p* = 0.062). Overall, improvement was observed in some of the management practices, but not up to the desired extent.

Compared to other crops, sorghum was dried in shorter durations across all three districts. This is because some of the sorghum produce was meant for the brewing industry and was sold quickly to earn income. Approximately 70% of the farmers stored maize produce at home for household consumption, while less than 15% stored maize in a warehouse. 

### 2.3. Aflatoxin Contamination in Crop Samples

At the baseline, 40%, 45% and 51% of grain samples of groundnut, maize and sorghum respectively were contaminated with aflatoxin at levels higher than 20 ppb (Table 5). The average contamination level was highest for groundnut (146.6 ppb), followed by sorghum (119.5 ppb). By the end-line, there was a 10% point, 13% point and 18% point reduction in the incidence of aflatoxin B_1_ (AFB_1_) contamination, with levels greater than 20 ppb in groundnut, maize and sorghum, respectively. At the end-line, 73% of the groundnut, 68% of the maize and 67% of the sorghum had less than 20 ppb of contamination. The results showed significant reduction in incidence of aflatoxin contamination above 20 ppb in maize (*p* = 0.045) and sorghum (*p* = 0.022), while the reduction for groundnut was statistically insignificant.

The crop samples that were matched between the baseline and end-line from the same households were analyzed (Table 6). The mean AFB_1_ level among the three crops was 83.6 ppb at the baseline and 55.8 ppb at the end-line. The change was found to be statistically significant using the Wilcoxon Signed Rank Test. There was an overall reduction in the proportion of the samples with AFB_1_ level above 20 ppb by 11.9% points. Judging from Table 5, the reduction has supposedly stemmed from maize and sorghum samples. 

Lastly, on average, farmers who had started adopting proper drying and grading practices had experienced significantly lower aflatoxin contamination in their produce at the end-line compared to the baseline (Table 7).

## 3. Discussion

The mean aflatoxin contamination level was high in groundnut compared to the produce of other crops, which is consistent with the findings from other studies [7,8]. This is probably due to the exposure of groundnut directly to the soil [7]. Maize and sorghum also had significant contamination, which was attributed to the airborne contamination. At first, farmers in this study tended to have limited knowledge, a negative attitude and inappropriate practice of pre- and post-harvest crop management. Following the training on aflatoxin contamination and crop management, there was some increase in knowledge, especially on grading. Some improvement in practices was also observed, especially in grading and storage of grains, particularly in Nsanje. 

In Nsanje, the production of groundnut was relatively high compared to other crops and to other districts. Similarly, in Chikwawa, the production of sorghum was relatively high compared to other crops and to other districts. The training on post-harvest management especially resulted in increasing the rate of adoption of the grading of these relatively important crops (i.e., groundnut in Nsanje and sorghum in Chikwawa). This emphasizes the importance of training on crop-specific pre-and post-harvest management practices. Other studies also reported that grading is a critically important step in mitigating aflatoxin contamination, especially when there are no other mitigation methods available [11]. Other studies indicate that physical sorting practice alone reduces aflatoxin contamination by 40–80% [17,18].

Moreover, farmers’ knowledge on mulching improved and they also found it useful in increasing soil moisture and crop yield, especially during the dry season following the 2017–2018 rainy season. As a result, they developed a positive attitude toward this technique. In this study, however, farmers’ attitude toward some critical management practices did not change significantly in spite of undergoing the training program on good practices and sensitization regarding the negative impacts of contaminated grade outs on health and economy. One such attitude was the limited willingness to discard grade outs even after learning of its negative impacts. The FGD revealed that such unwillingness was due to the fact that the portion of grade outs accounted for 10% to 20% of their profit, and they could not afford to discard it. Although the results showed some reduction in the consumption of grade outs after the training, the farmers basically kept on selling them in markets, which entered the food supply chain. Another important key step was proper drying methods. Although farmers’ knowledge increased on proper drying methods, it was not adequately practiced due to space limitations at homesteads and the fear of theft in fields. These are the observed reasons why their attitude toward the recommended post-harvest practices did not change significantly. This could also explain why contamination largely remained at the end-line. Further, another unadvisable practice that invites contamination and its spread is sprinkling water to soften the groundnut shell. Despite the training, this practice did not change significantly. Admittedly, the practice of sprinkling water was the easiest way for women farmers to shell groundnut, compared with shifting to mechanical shelling unless they had such facilities at the community level [19].

Under such circumstances, it is vitally important to have affordable alternate methods to mitigate aflatoxin contamination. One possible example is oil extraction with subsequent alkali refining, washing and bleaching to reduce aflatoxin contamination in groundnut oil [20], though it is not suitable for all crops, and the availability of such facilities and the cost efficiency in rural settings are a challenge. Another possible method is the use of aflatoxin-binding agents to reduce contamination in food and feed. However, again, this is not readily available in Malawi [21].

Our FGDs clearly revealed cost implications for farmers in terms of buying consumables or adopting machinery. The economic implications for farmers in controlling aflatoxin seem to discourage them from following some of the appropriate practices. Therefore, aflatoxin contamination needs to be resolved through simple cost effective methods. A study conducted in Congo shows that farmers’ willingness to pay for improved practices was very low [22]. It is also important to note that currently there is no additional premium that farmers receive for selling clean and quality grains. Thus, expectations of behavioral change to reduce aflatoxin contamination may not be satisfied unless there is a clear economic benefit for farmers such as a price premium for quality grains. Hence, mitigation policies and initiatives should pay more attention to ensuring economic incentives for farmers to deliver quality grains to markets, in parallel to enforcing training and demanding quality grains. Indeed, some practices such as mulching, drying and grading techniques are relatively easy to follow. However, another fatal issue was the continued use of grade outs. There is an urgent need for creating a complete intervention package that helps in reducing aflatoxin contamination without compromising farmers’ needs and incentives.

Although some levels of aflatoxin were detected in most of the crop samples even after the training, the levels of contamination reduced significantly despite the heavy rain and flood that occurred in the target areas. The result implies the effectiveness of the proper practices undertaken following the training such as drying and grading during the 2017–2018 season, which is consistent with our recent finding in Tanzania [23].

The present study has a few limitations. First, there was no control group in this study. Without surveying farmers who received neither the training nor its spillover, the observed changes in the intervention group alone cannot fully be attributed to the training program, if the assumption of no external influence is violated. Second, there was attrition in the crop samples from the baseline to end-line which may have resulted in some bias in the result if the attrition had occurred non randomly. The attrition in crop samples was mainly due to the flood that affected the target regions and caused some crop failure, raising food insecurity concerns and unwillingness to supply grain samples. Third, aflatoxin detection was performed only with fresh grain samples but not with storage samples, which was partly due to the low harvest and not enough food grains to be stored during the baseline and end-line period. A similar study on storage samples would contribute to evidence of aflatoxin contamination and mitigation at the most critical stage of post-harvest processes before and after training.

## 4. Conclusions

The current study suggests that it is possible to reduce aflatoxin levels in crop produce when mitigation measures are integrated and if such measures are practiced strictly. Although training helps farmers acquire knowledge, it is also important to address their attitude toward the mitigation practices by recommending mitigation measures that are simple and affordable. Otherwise, farmers will continue to be reluctant to practice the mitigation measures despite noting the negative health and overall economic effects of aflatoxin contamination in their agricultural produce.

## 5. Materials and Methods 

### 5.1. Participants in the KAP Studies

Nine Extension Planning Areas (EPAs) were purposively selected from three districts in Southern Malawi as the study site where significant quantities of maize, groundnut and sorghum are produced and consumed, namely, Lirangwe, Kunthembwe, and Lunzu EPAs in Blantyre; Kalombe, Livuzi, and Mitole EPAs in Chikwawa; and Makhanga, Nyachilenda, and Zunde EPAs in Nsanje. 

Purposive sampling was used to choose farmers producing groundnut, maize and/or sorghum, from which random sampling drew 900 households for the baseline survey conducted in May to June 2017. During the 2017–2018 crop season, 420 randomly selected farmers received training from trainers and then in turn initiated a process of training the rest of the farmers. In other words, it was expected that all the 900 farmers received either the training of trainers (i.e., direct training) or the training (i.e., indirect training). This method of dissemination of agricultural knowledge and practices was justified in the previous study conducted by Nakano et al. [24]. While our research did not use a control group, it was closely monitored in each EPA through the crop officers to ensure that there was no external influence on the farmers’ practice during the study period other than our intervention and its spillover. Therefore, this study assumes that the before-after comparison would serve as the with-without comparison. After the training, 624 out of the initial pool of 900 households participated in the end-line survey conducted in May to June 2019. This reduction in sample size is basically due to migration, crop failure due to flood and farmers’ mere reluctance to participate at the end-line. A semi-structured questionnaire was programmed with Open Data Kit (ODK) to electronically collect data from the farmers in order to understand the KAP regarding pre- and post-harvest crop handling methods and aflatoxin contamination. 

While the surveys largely focused on the knowledge and practice components of KAP, ten focus group discussions were conducted with 20–25 farmers per group to elicit farmers’ attitude toward improved methods and gain clarity on why farmers persisted with unadvisable practices despite knowing their negative effects. In other words, the study applied the mixed methods to capture the three components of KAP regarding aflatoxin contamination and mitigation measures.

### 5.2. Grain Sample Collection

From the 900 households at the baseline, we collected 631 freshly harvested grain samples of groundnut, 127 of maize, and 87 of sorghum. Not all farmers managed to provide grain samples mainly because of a food insecurity concern despite the willingness of the research team to monetarily compensate for the grains. At the end-line, from the 624 farmers, we collected a total of 696 samples of groundnut, maize, and sorghum to test for AFB_1_ (Table 8). 

From each farmer, sub-samples were collected from multiple depths in each harvested bag and then pooled into a single sample of approximately 2 kg. We then took 500 g from the pooled sample as the composite sample to be analyzed, which was kept in paper bags [25]. The samples were later air dried and transported within a week to the laboratory and stored at 5 °C until assayed for aflatoxin contamination. 

### 5.3. Quantitative Detection of Aflatoxin B1 from Grain Samples 

A 100 g sample was weighed from each 500 g sample collection and milled into powder—from which, two analytical samples of 20 g each were each mixed with 100 mL of 70% methanol (v/v), augmented with 0.5% potassium chloride (KCl) and blended further. The mixture was then transferred to a 250 mL conical flask and shaken at 300 rpm for 30 min (Gallenkamp Orbital Shaker, CAT # SCM 300 0101, England), and filtered through Whatman No. 41 filter paper (GE Healthcare, Buckinghamshire HP7 9NA, UK). The filtrate was assayed for aflatoxin using Indirect Competitive Enzyme Linked Immunosorbent Assay (IC-ELISA) using a 96 well ELISA plate (F96 MAXISORP, Thermo Fisher Scientific, Denmark) with a detection limit of 1 ng/g [7,26]. In brief, the samples were tested using polyclonal antibody produced against AFB_1_ [7,26]. Alkaline phosphatase conjugated anti-rabbit antibodies (Sigma-Aldrich, St. Louis, USA) were used as a secondary antibody and para-nitrophenyl phosphate (pNPP) (Sigma-Aldrich, St. Louis, USA) used as a substrate. The colorimetric reaction was measured in an ELISA plate reader (multiscan reader, Thermo Fisher Scientific, China) using a 405 nm filter. To confirm the presence of aflatoxin in a selected sample, the filtrate was subjected to thin layer chromatography (TLC) using silica gel-coated 20 × 20 cm glass plates (Fluka Analytical, Sigma-Aldrich, St. Louis, USA) and visualized under UV light [27]. 

### 5.4. Training on Pre- and Post-Harvest Crop Management

After the baseline survey, the training was conducted during the 2017–2018 crop growing season on aflatoxin contamination, its hazard and its mitigation through pre and post-harvest crop management. The participatory approach was followed in training farmers by providing them with hands-on training in the field and using supporting materials including the materials prepared in both English and Chichewa (local language spoken in the study area) and the sample demonstration materials. 

The key questions addressed during the training include: what is aflatoxin? What are its effects on health and economy? How does contamination occur? And how can we mitigate it? The training covered a wide range of agronomic practices from crop rotation to the timing of planting, plant spacing, soil amendments, water management, tied ridges, mulching, irrigation, weeding, harvesting, shelling, drying, grading, storage and transportation. 

### 5.5. Statistical Analysis 

Data collected during the baseline and end-line surveys were cleaned, validated, organized, coded, and subjected to statistical analysis using STATA version 14 [28]. Descriptive statistics such as frequency, mean, median and standard deviation were used to present the knowledge and practices among farmers and aflatoxin contamination levels in crop samples. Inferential statistics such as the chi square test, Wilcoxon signed rank test and Mann–Whitney U test were performed to examine the statistical significance in the changes that have resulted. The purpose of using the non-parametric statistics was to address the non-normal distribution of aflatoxin contamination levels.

## Figures and Tables

**Table 1:** Farmers’ knowledge on aflatoxin contamination at different stages of crop production (baseline vs. end-line): Percentage of the respective response, the change, and its statistical significance (*n* = 306).

Statement	% of Farmers in Agreement			
	Baseline (%)	End-Line (%)	Increase in % Point	χ^2^ (*p*)
Contamination begins in the field	66.2	77.7	11.5	0.433
Contamination occurs during harvest	73.0	87.6	14.7	0.503
Contamination increases due to improper drying	75.7	90.1	14.4	0.817
Contamination occur during crop storage	82.4	89.6	7.2	0.144
Contamination spreads if the crop produce is not graded	60.1	93.1	29.9	0.851
Contamination occurs if the storage place is wet	90.5	96.5	6.0	0.527
Contamination increases if water is sprinkled during shelling	83.1	85.2	2.0	0.717
Contamination increases if water is sprinkled to increase weight	77.7	83.2	5.5	0.674
The consumption of contaminated grains causes child stunting	31.8	68.3	36.6	0.577
Aflatoxin-contaminated grains will be rejected in the market	66.9	87.1	20.2	0.551
Aflatoxin contamination causes income loss	71.6	90.6	19.0 *	0.092
Contaminated grains should not be fed to livestock	37.8	72.3	34.4 *	0.075
Aflatoxin-contaminated grains reduce livestock productivity	37.8	70.8	33.0 **	0.033

** and * indicate the statistical significance of the change, corresponding to *p* < 0.05 and *p* < 0.10, respectively, according to the Chi square (χ^2^) test.

**Table 2:** Attitude of the farmers toward some of the aflatoxin management practices: Quotes from the focus group discussions.

Statements/Questions Asked toward Farmers Attitude on Pre- and Post-Harvest Crop Practice	Farmer’s Answer
Why do you still consume grade out/why do you still feed grade out/why do you sell grade out in market?	“This is our 10 to 20 percentage of income, which can’t be thrown away.”
	“We don’t have enough food during these bad seasons; so we can’t throw them away.”
	“What do I do with grade outs? because it is money or food, and can you teach some other methods to clean them from contamination?”
	“We are eating them since childhood; we are still fine.”
Why do you not dry your grains on raised bed/tarpaulin/ground cover?	“We don’t have enough space.”
	“We can’t spread them in farm because we will lose them to thieves.”
	“I don’t want to spend in buying tarpaulin.”
Why do you still sprinkle water to shell the groundnut?	“It makes it easy to shell.”
	“I don’t keep them for a long time. I sell them immediately, so this practice doesn’t affect me.”
Why do you continue mulching practice?	“It is simple.”
	“It holds moisture, so my crops doesn’t dry out during drought and I get good yield.”
	“It helped for two to three weeks of no rain during this season.”

**Table 3:** Grading practice of the farmers in the three districts of Malawi (baseline vs. end-line): percentage of farmers practicing grading, the change, and its statistical significance (*n* = 306).

	Blantyre				Chikwawa				Nsanje			
Crops	Baseline (%)	End-Line (%)	Change in % Point	χ^2^ (*p*)	Baseline (%)	End-Line (%)	Change in % Point	χ^2^ (*p*)	Baseline (%)	End-Line (%)	Change in % Point	χ^2^ (*p*)
Maize	92.0	90.4	1.6	0.651	71.2	85.2	14.0	0.890	54.8	70.5	15.7	0.355
Groundnut	92.9	76.9	−15.9	0.263	73.5	88.9	15.4	0.786	58.7	86.1	27.4**	0.012
Sorghum	57.7	71.4	13.7	0.273	46.7	62.5	15.8*	0.097	47.1	37.5	9.6	0.996

** and * indicate the statistical significance of the change, corresponding to *p* < 0.05 and *p* < 0.10, respectively, according to the Chi square (χ^2^) test.

**Table 4:** Farmers’ post-harvest practices (baseline vs. end-line) (*n* = 306).

Question/Statement on Farmers Practice	Practice on All Crops			
	Baseline (%)	End-Line (%)	Change in % Point	χ^2^ (*p*)
Dry produce on bare soil/roof	50.7	39.5	11.1	0.182
Dry produce on tarpaulin sheet/mat	70.9	95.8	24.8	0.166
Shell the groundnut pod/cob using machine	0.7	1.3	0.7	0.870
Shell the groundnut pod/cob manually	76.8	99.7	22.9 *	0.068
Sprinkle water for shelling groundnut	6.6	10.2	3.6 ***	0.004
Grade grains	62.4	92.5	30.1	0.873
Grade grains to improve quality	60.8	89.9	29.1	0.653
Grade grains to separate them based on color and size	31.1	35.3	04.2	0.891
Throw away grade outs	44.4	60.1	15.7 *	0.090
Feed grade outs to livestock	10.5	7.5	2.9	0.774
Consume grade outs in different forms	51.0	45.1	5.9 ***	0.009
Sell grade outs in markets	0.7	3.9	3.3	0.774
Store them in gunny bag/polythene bag	26.1	26.5	0.3 *	0.086
Use hermetic storage	0.0	2.9	2.9	NA
Store bags on wooden palates	60.8	78.8	18.0 *	0.062
Store bags on floor	12.4	24.1	12.8	0.630

*** and * indicate the statistical significance of the change, corresponding to *p* < 0.01 and *p* < 0.10, respectively, according to the Chi square (χ^2^) test; NA: *p*-value not identified.

**Table 5:** Aflatoxin contamination levels by crop in all three districts (baseline vs. Eend-line) (*n* = 696).

Indicator	Groundnut		Maize		Sorghum	
	Baseline	End-line	Baseline	End-line	Baseline	End-line
Number of samples tested (*n*)	589	386	114	268	79	42
% of samples that tested negative for AFB_1_	31.4	2.8	49.1	2.9	30.3	4.7
% of samples that tested positive and with < 20 ppb of AFB_1_	55.4	70.4	55.1	65.0	49.0	62.5
% of samples with > 20 ppb of AFB_1_	39.6	29.6	44.8	31.9	50.9	32.5
Median AFB_1_ (ppb)	1.97	7.92	0.00	7.22	0.09	10.42
Mean AFB_1_ (ppb)	146.6	56.6	25.8	43.8	119.5	103.7
Standard Deviation AFB_1_ (ppb)	1929.9	173.3	142.0	144.3	245.4	296.3
Percentage point reduction in contamination level > 20 ppb	10.0		12.9		18.4	
*p*-value for Chi square (χ^2^) test	0.886		0.045		0.022	

**Table 6:** Aflatoxin contamination levels in samples of all three crops (*n* = 416).

Indicator	Baseline	End-Line
Number of samples tested (*n*)	416	548
% of samples that tested negative * for AFB_1_	37.6	18.4
% of samples that tested positive and with < 20 ppb of AFB_1_	54.9	66.8
% of samples with > 20 ppb of AFB_1_	45.1	33.2
Median AFB_1_ (ppb)	1.0	9.1
Mean AFB_1_ (ppb)	83.6	55.8
Standard Deviation AFB_1_ (ppb)	297.2	179.0
Wilcoxon Signed Rank Test (*p*-value)	0.001	
Reduction in incidence of contamination level > 20 ppb (% points)	11.9	

* less than the detection limit of 1 ng/g.

**Table 7:** AFB_1_ levels in grain samples of the farmers who adopted the particular practice after the training (*n* = 383).

Method Adopted after the Training	AFB_1_ Level in Grain Samples (ppb)		Mann–Whitney U Test	
	Baseline	End-Line	U-Statistic	*p*-Value
Grading				
Mean	73.3	50.9	65.09	0.000
SD	260.8	164.7		
Drying their harvest on tarpaulin sheets/mats cover				
Mean	72.1	55.9	55.62	0.001
SD	261.5	178.4		

SD: standard deviation.

**Table 8:** Number of sampled households and crop samples collected by crop and by district for the baseline and end-line.

District	Number of Sampled HHs		Crop Samples	Number of Crop Samples Collected	
	Baseline	End-Line		Baseline	End-Line
Blantyre	305	196	Groundnut	224	149
			Maize	21	57
			Sorghum	5	0
			Subtotal	250	206
Chikwawa	314	237	Groundnut	130	93
			Maize	56	118
			Sorghum	47	29
			Subtotal	233	240
Nsanje	281	191	Groundnut	173	144
			Maize	40	93
			Sorghum	30	13
			Subtotal	243	250
Total	900	624	Grand total	726	696
